# Determining optimal gestational weight gain in the Korean population: a retrospective cohort study

**DOI:** 10.1186/s12958-017-0280-3

**Published:** 2017-08-22

**Authors:** Sae Kyung Choi, Guisera Lee, Yeon Hee Kim, In Yang Park, Hyun Sun Ko, Jong Chul Shin

**Affiliations:** Department of Obstetrics and Gynecology, College of Medicine, The Catholic University of Korea, 222, Banpo-daero, Seocho-gu, Seoul, 06591 Republic of Korea

**Keywords:** Pregnancy, Weight gain, Pregnancy outcomes, Body mass index

## Abstract

**Background:**

The World Health Organization (WHO) international body mass index (BMI) cut-off points defining pre-pregnancy BMI categories in the Institute of Medicine (IOM) guidelines are not directly applicable to Asians. We aimed to define the optimal gestational weight gain (GWG) for the Korean population based on Asia-specific BMI categories.

**Methods:**

Data from 2702 live singleton deliveries in three tertiary centers between 2010 and 2011 were analyzed retrospectively. A multivariable logistic regression analysis was conducted to determine the lowest aggregated risk of composite perinatal outcomes based on Asia-specific BMI categories. The perinatal outcomes included gestational hypertensive disorder, emergency cesarean section, and fetal size for gestational age. In each BMI category, the GWG value corresponding to the lowest aggregated risk was defined as the optimal GWG.

**Results:**

Among the study population, 440 (16.3%) were underweight (BMI < 18.5), 1459 (54.0%) were normal weight (18.5 ≤ BMI < 23), 392 (14.5%) were overweight (23 ≤ BMI < 25) and 411 (15.2%) were obese (BMI ≥ 25). The optimal GWG by Asia-specific BMI category was 20.8 kg (range, 16.7 to 24.7) for underweight, 16.6 kg (11.5 to 21.5) for normal weight, 13.1 kg (8.0 to 17.7) for overweight, and 14.4 kg (7.5 to 21.9) for obese.

**Conclusion:**

Considerably higher and wider optimal GWG ranges than recommended by IOM are found in our study in order to avoid adverse perinatal outcomes. Revised IOM recommendations for GWG could be considered for Korean women according to Asian BMI categories. Further prospective studies are needed in order to determine the optimal GWG for the Korean population.

## Background

The rates of overweight and obesity among women of childbearing age have risen dramatically, and this represents a medically important issue [1, 2]. The prevalence of obesity among women in Korea is 27.5%, and this rate increases to 47.8% when the overweight and obese categories are combined. From 1998 to 2001, the prevalence of obesity increased from 25.9% to 29.1% in Korean women. According to recent data from the Korea Centers for Disease Control and Prevention, the Korea National Health and Nutrition Examination Survey, and Ministry of Health and Welfare, this increase has leveled out, with no significant change in the prevalence of obesity among Korean women between 2005 and 2013. However, the rate of obesity increases rapidly with age in childbearing women. When we consider the increased prevalence of older age pregnant women in Korea, the proportion of obese childbearing-aged women is of greater concern, despite the fact that recent data do not show a significant increase in obesity among Korean women.

Obese women tend to gain weight during pregnancy excessively, resulting in postpartum weight retention. These women will not only have a high risk pregnancy due to the pre-pregnancy obese state, but will also have a high risk for metabolic disorders in the future [3]. Moreover, excessive gestational weight gain causes maternal and neonatal complications, such as gestational diabetes, hypertensive disorder, labor induction, cesarean delivery, anesthetic complications, postpartum hemorrhage, neonatal intensive care unit admission, macrosomia, and congenital anomalies [4–8]. Therefore, proper gestational weight gain is important for improving perinatal outcomes.

The Institute of Medicine (IOM) suggested new guidelines for adequate gestational weight gain in 2009, considering the incidences, long-term sequelae, and baseline risks of several potential outcomes associated with gestational weight gain. The new guidelines specified different weight gains for women who were underweight, normal weight, overweight, and obese. These classifications are based on body mass index (BMI), defined as weight in kilograms divided by height in meters squared (kg/m2). The IOM guidelines recommend a weight gain of 12.5–18 kg for the underweight group (BMI < 18.5), 11.5–16 kg for the normal group (18.5 ≤ BMI < 25), 7–11.5 kg for the overweight group (25 ≤ BMI < 30), and 5–9.1 kg for the obese group (BMI ≥ 30) [9].

The IOM guideline is most widely used because it is applicable to various racial and ethnic groups. However, this guideline is mainly based on the Caucasian standard, and confirmatory studies are needed because there may be racial differences in body conditions, such as maternal height, pelvic shape, and fat deposition according to weight gain. Furthermore, the World Health Organization (WHO) expert consultation revised the cut-off value of BMI to determine overweight and obesity in the Asian population. WHO expert consultation discussed this issue on the grounds that Asians have a different correlation between BMI, body fat deposition, and health risk than Europeans [10]. Asians are more likely to have a lower BMI, even though they tend to have more abdominal obesity than other races. Furthermore, in Asia, the risk of type 2 diabetes and cardiovascular disease is higher in person with BMI < 25 [11]. Therefore, a revised recommendation for gestational weight gain is needed for Asian people.

The purpose of the present study is to suggest the proper gestational weight gain (GWG) considering Asian population-specific characteristics. We aimed to define GWG ranges for each pre-pregnancy BMI category defined by the WHO Asian classification among Korean women.

## Methods

From 2010 to 2011, 4557 pregnant women delivered their babies in three tertiary centers at the Catholic Medical Center, Seoul St. Mary’s Hospital, Uijeongbu St. Mary’s Hospital, and St. Vincent Hospital. We retrospectively reviewed the medical records of 3285 term (37 completed weeks of gestation or later) singleton pregnant women. The pre-pregnancy BMI and GWG were calculated using pre-pregnancy body weight reported by each individual and physical measurement at admission for delivery. We excluded persons that are not Koreans or did not report their body weight before pregnancy. Finally, 2702 pregnant women were enrolled as the study population.

We reviewed pre-pregnancy BMI, weight gain during pregnancy, and maternal and neonatal outcomes in the medical records. The maternal outcomes included gestational hypertensive disorder, gestational diabetes, mode of delivery, emergency cesarean section due to failed labor, 4th degree perineal laceration, and postpartum hemorrhage and infection. The neonatal outcomes included fetal size for gestational age.

The pre-pregnancy BMI was classified according to Asia-specific standards from the WHO as follows:

1. Underweight: BMI < 18.5
2. Normal: 18.5 ≤ BMI < 23
3. Overweight: 23 ≤ BMI < 25
4. Obese: BMI ≥ 25

The size of the neonate was based on their birth weight at delivery. Birth weight that was less than the 10th percentile was classified as small for gestational age (SGA) and birth weight greater than the 90th percentile was classified as large for gestational age (LGA). This was derived from the most widely used criterion based on a worldwide study conducted Alexander GR et al. [12].

Statistical analyses were performed using SAS version 9.3 (SAS Institute Inc., Cary, NC, USA). Discrete data are expressed as number (%) by analysis of the chi-square test. Continuous data are expressed as mean ± standard deviation or median values using ANOVA or Kruskal-Wallis test respectively. We confirmed the perinatal outcomes that showed meaningful changes according to GWG. The most meaningful perinatal outcomes included gestational hypertensive disorder, emergency cesarean section, and fetal size for gestational age. A univariate multivariable logistic regression analysis was conducted to determine odd ratios (with 95% confidence intervals [CI]) of each outcome relating to increase in GWG. They were adjusted for age, parity, occupation, mode of delivery, and medical history as factors affecting GWG. Total risk was estimated by spline estimation using predicted risk for each complication. We defined the GWG ranges stratified according to Asia-specific BMI categories that did not exceed a 5% increase from the lowest predicted risk.

This study was approved by ethics committee of the Clinical Research Coordinating Center of the Catholic Medical Center (XC11RIMI0029K).

## Results

Baseline characteristics and obstetric outcomes of study participants are summarized in Table 1. Among the study population, 440 (16.28%) were underweight (BMI < 18.5), 1459 (54.00%) were normal (18.5 ≤ BMI <23), 392 (14.47%) were overweight (23 ≤ BMI < 25), and 412 (15.25%) were obese (25 ≤ BMI). Half of the study population was primiparous, and the other half was multiparous. One thousand nine hundred fifty five participants (59.18%) underwent vaginal delivery, and 1103 (40.82%) underwent cesarean section. Among the individuals who underwent cesarean section, 266 underwent operation due to emergency such as arrest disorder or non-reassuring fetal heart rate, defined as failed labor. The rates of gestational diabetes and gestational hypertensive disorder were 6.22% and 5.03%, respectively.

**Table 1:** Baseline characteristics of the study population

Characteristics		Total = 2702
Age (years)		32.59 ± 4.75
Occupation	Yes	981 (36.31%)
	No	1721 (63.69%)
Education	Elementary school or less	19 (0.70%)
	Middle school graduate	69 (2.55%)
	High school graduate	893 (33.05%)
	University graduate or higher	1821 (63.69%)
Medical history	Yes	350 (12.95%)
	No	2352 (87.05%)
Pre-pregnancy BMI category	Underweight (< 18.5 kg/m^2^)	440 (16.28%)
	Normal (18.5 to <23 kg/m^2^)	1459 (54.00%)
	Overweight (23 to <25 kg/m^2^)	391 (14.47%)
	Obese (≤ 25 kg/m^2^)	412 (15.25%)
Parity	Primiparous	1351 (50%)
	Multiparous	1351 (50%)
Duration of pregnancy (weeks)		38.73 ± 1.29
Mode of delivery	Vaginal delivery	1599 (59.18%)
	Cesarean section	1103 (40.82%)
	elective	837 (30.98%)
	emergency	266 (9.84%)
Size for gestational age	SGA	358 (13.25%)
	AGA	2217 (82.05%)
	LGA	127 (4.7%)
Gestational diabetes		168 (6.22%)
Gestational hypertensive disorder		136 (5.03%)

*BMI* body mass index, *SGA* small for gestational age, *AGA* appropriate for gestational age, *LGA* large for gestational age

### Perinatal outcomes

**Fig. 1:**
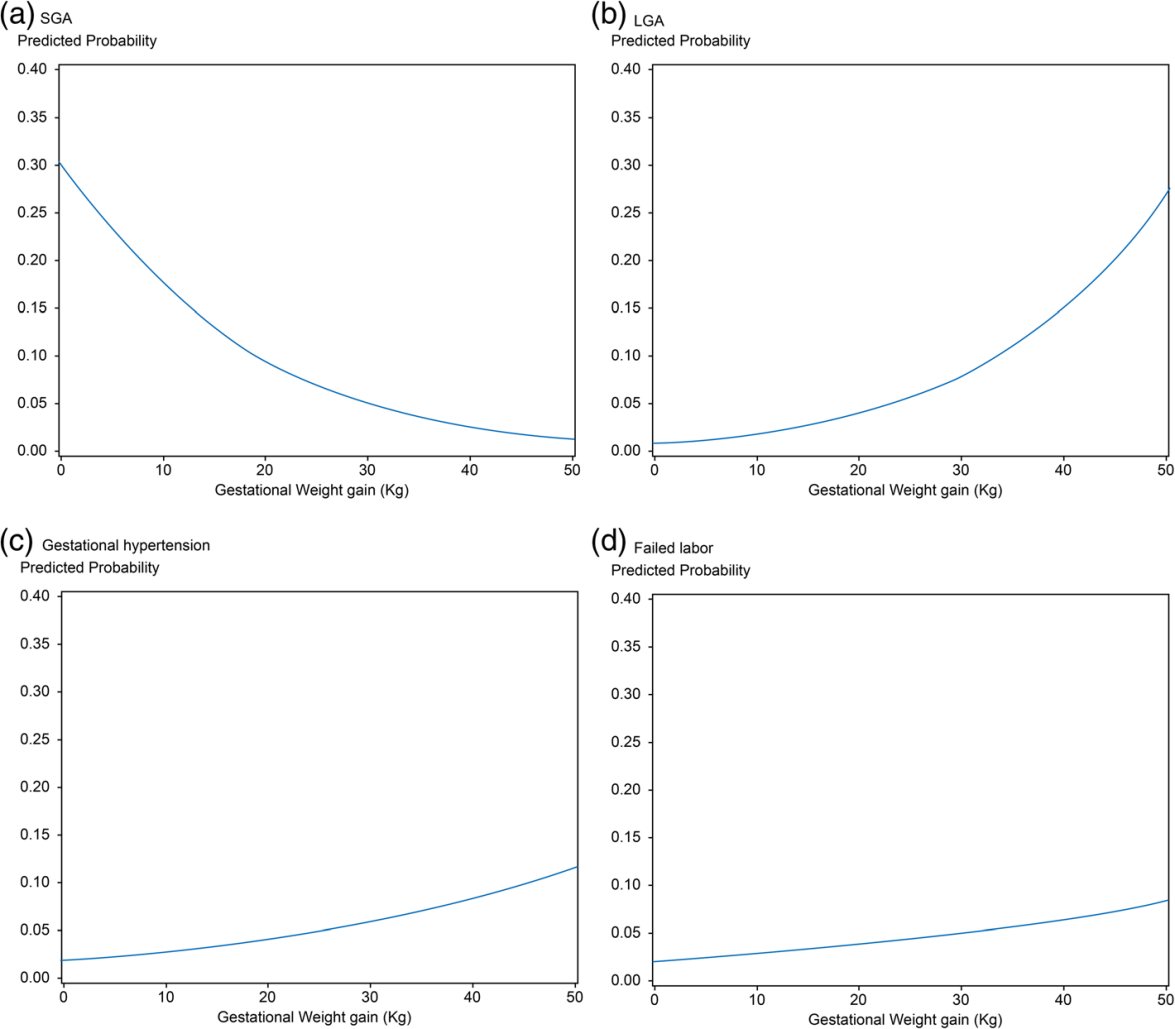
Predicted risk of each composite outcome. **a** The predicted risk of SGA with GWG. **b** The predicted risk of LGA with GWG. **c** The predicted risk of gestational hypertension with GWG. **d** The predicted risk of failed labor with GWG. GWG was positively correlated with the predicted risk of LGA, gestational hypertension, and failed labor, but showed a negative correlation with the predicted risk of SGA. *SGA*, small for gestational age; *LGA*, large for gestational age; *GWG*, gestational weight gain

Perinatal outcomes indicating significant risks with a GWG of 1 kg were gestational hypertensive disorder, emergency cesarean section, and fetal size for gestational age. Logistic models for perinatal outcomes showed that an increase in GWG was associated with a decrease in the predicted risk of SGA and increase in that of LGA. Furthermore, increase in GWG was associated with increased predicted risks of gestational hypertension and failed labor (Fig. 1).

The odd ratio for each outcome was calculated on the basis of maternal age, height, parity, occupation, education, medical history, and mode of delivery. All confounders were highly significant predictors of GWG. The risk for SGA decreased (OR 0.93; 95% CI = 0.91–0.96) and the risk for LGA increased (OR 1.07; 95% CI = 1.04–1.11) as the GWG increased by 1 kg. The incidence of gestational hypertension increased according to GWG (OR 1.03; 95% CI = 1.00–1.07). The risk of failed labor also increased (OR 1.02; 95% CI = 0.99–1.05), but the adjusted odd ratio was not statistically significant (Table 2).

**Table 2:** Odds of each outcome relating to one unit increase in GWG

	Unadjusted		Adjusted	
	OR (95% CI)	*P* value	OR (95% CI)	*P* value
SGA	0.93 (0.91–0.96)	<0.0001	0.93 (0.91–0.96)	<0.0001
LGA	1.08 (1.05–1.12)	<0.0001	1.07 (1.04–1.11)	<0.0001
Gestational hypertension	1.04 (1.00–1.07)	0.0327	1.03 (1.00–1.07)	0.0601
Failed labor	1.05 (1.02–1.07)	<0.0001	1.02 (0.99–1.05)	0.1273

Adjusted for age, height, parity, occupation, education, medical history, and mode of delivery

*GWG* gestational weight gain, *OR* odds ratio, *CI* confidence interval, *SGA* small for gestational age, *LGA* large for gestational age

### Gestational weight gain

**Fig. 2:**
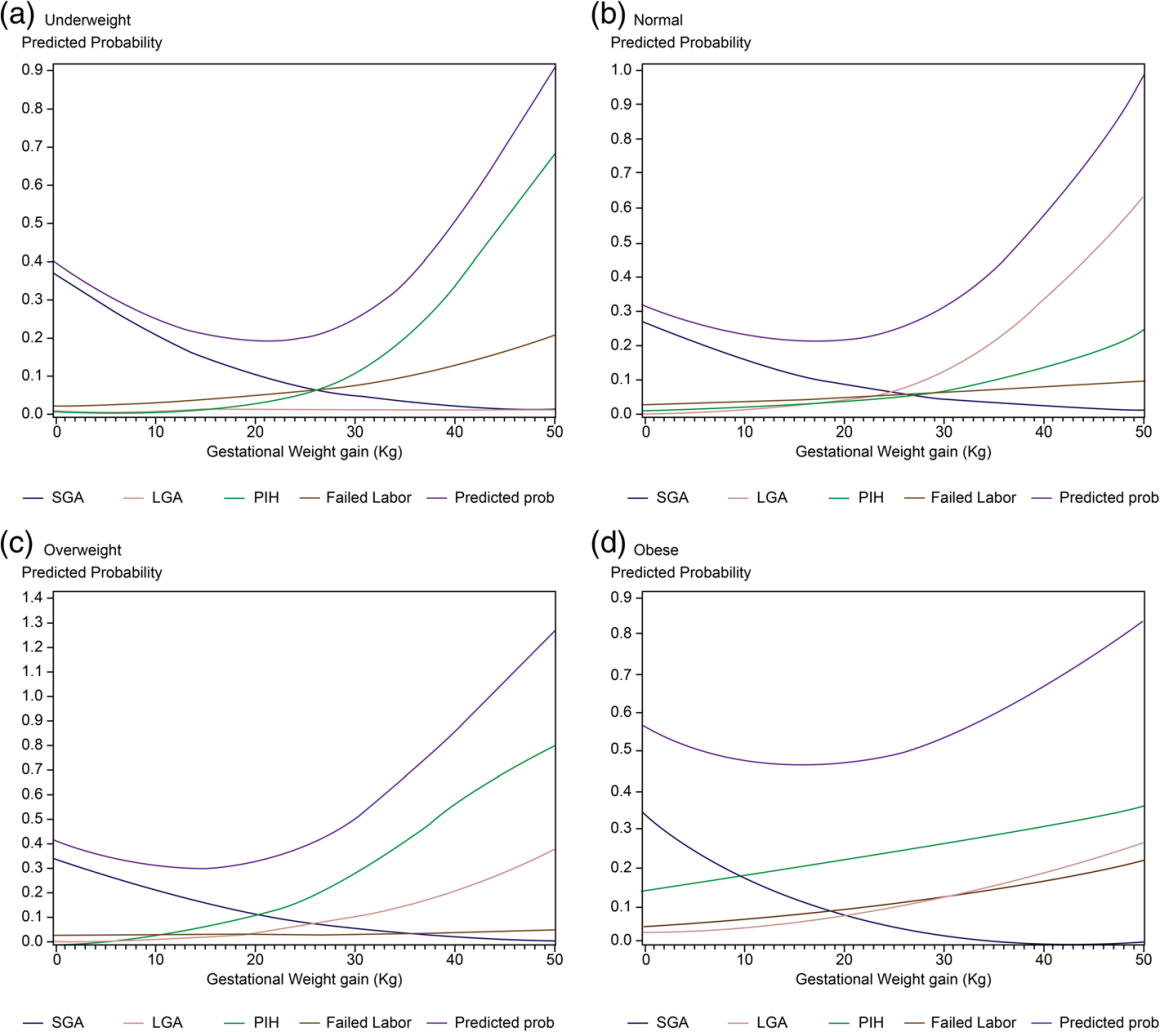
Predicted risk by body mass index classification. **a** Underweight group. **b** Normal group. **c** Overweight group. **d** Obese group. Total predicted risks were calculated using the risk for each complication. We defined the lowest total predicted risks and recommended weight gain ranges that did not exceed a 5% increase from the lowest predicted risks according to Asia-specific BMI categories. *BMI*, body mass index; *SGA*, small for gestational age; *LGA*, large for gestational age; *PIH*; pregnancy induced hypertension

The lowest total predicted risks were calculated in each interval according to the Asian BMI classification. The recommended weight gain range was set as the range that does not exceed a 5% increase from the lowest predicted risk. The optimal GWG values were observed to be between 16.7 and 24.7 kg for underweight women and between 11.5 and 21.5 kg for normal women. Overweight and obese women achieved the optimal GWG values between 8.0 and 17.7 kg and 7.5 and 21.9 kg, respectively (Table 3). The optimal GWG rages were considerably higher and wider than GWG reported in the IOM guideline. Furthermore, the optimal GWG for obese group was wider than that for overweight group. Because the pre-pregnancy obesity itself was an important risk factor for adverse perinatal outcome, it was not easy to determine the proper range of GWG in the obese group, thus suggesting a broad and vague GWG range (Fig. 2).

**Table 3:** Optimal weight gain range with the lowest risk

Pre-pregnancy weight category	Asian Pacific BMI	Optimal weight gain range (kg)
Underweight	Less than 18.5 kg/m^2^	20.8 (16.7–24.7)
Normal	18.5–22.9 kg/m^2^	16.6 (11.5–21.5)
Overweight	23.0–24.9 kg/m^2^	13.1 (8.0–17.7)
Obese	25 kg/m^2^ and greater	14.4 (7.5–21.9)

*BMI* body mass index

## Discussion

In this study, we examined the proper weight gain range during pregnancy according to Asian BMI classification. When inappropriate weight gain was observed, the incidence of poor perinatal prognostic factors such as LGA, hypertension, emergency cesarean section, and SGA increased. Optimal GWG based on the risk of meaningful perinatal outcomes was higher for women who were in a lower BMI category and lower for women who were in a higher BMI category. The optimal GWG in this Korean population differed from that of the IOM guidelines, which was based on data from Caucasian women. The optimal GWG in our study was higher and the range was wider than that of the IOM guideline. The optimal GWG for underweight and obese women was outside the IOM recommended range.

Several previous studies have found the importance of proper GWG. Not only excessive GWG, but also insufficient weight gain can cause poor perinatal outcomes. While excessive weight gain during pregnancy is associated with LGA, maternal hypertension, and cesarean section, low maternal weight or insufficient GWG is associated with intrauterine fetal growth restriction, low birth weight, and preterm delivery [13–15]. Bodnar et al. explored the association between GWG and SGA, LGA, spontaneous preterm births (PTB), and medically indicated PTBs among 5550 pregnant women. They reported that the adjusted risk of SGA increased as GWG declined and the risk of LGA increased with increasing GWG. They also reported that low GWG were associated with an increased risk of spontaneous PTB and high GWG was related to an increased risk of indicated PTB [13]. Kiel et al. reported a population-based cohort study of 20,251 pregnant obese women delivering full-term singleton infants to examine the effect of GWG on pregnancy outcomes (preeclampsia, cesarean delivery, SGA, and LGA). They reported that increasing risk of preeclampsia, cesarean delivery, and LGA birth and decreasing risk of SGA birth with increasing GWG [15]. The results of our study were similar to these findings.

We suggested an optimal GWG guideline based on Asian BMI categories that differed from the IOM guideline. Some previous studies provided insight into whether racial or ethnic differences altered the association between GWG and various perinatal outcomes. The results from theses analyses were that racial or ethnic group did not affect the relationship between GWG and outcomes [16, 17]. Nevertheless, in some studies, various recommendations for different populations were proposed besides the IOM GWG guideline. The Brazilian Ministry of Health recommended its own GWG guideline based on the IOM guidelines and Atalah’s curve, which was a recommendation for Chilean women to monitor the progress of their nutritional state during pregnancy according to pre-pregnancy BMI [18, 19]. There were also several studies that suggested optimal GWG for Asian populations such as China, Vietnam, and Singapore [20–22]. This implies that the GWG should be different depending on demographic characteristics.

The optimal GWG range in our study was higher and wider than the IOM recommendation, as reported by Andreas et al. They established a GWG guideline using a new statistical technique by setting the GWG as a continuous variable and considering factors that affect weight gain, such as smoking or parity, as effect modifiers [23]. We hypothesize that the reason for the wider and higher GWG is not only differences in statistical methods but also the lower proportion of LGA in the study population limited to Korean women showing minimal ethnic differences.

The optimal GWG range for obese women was similar to that for overweight women but wider than those for other groups because predicted risks for all factors were relatively higher regardless of weight gain. This result supports our previous finding that pre-pregnancy obesity itself is a major risk factor for adverse perinatal outcomes. We previously reported that pre-pregnancy obesity more closely correlated with adverse perinatal outcomes than excessive GWG [24].

In summary, the most notable prognostic factors related to GWG were inappropriate birth weight, development of gestational hypertensive disorder, and increasing rate of emergency cesarean section. Our study indicates that GWG guidelines should be revised based on the characteristics of the Korean population considering these factors. Our guideline suggests an optimal GWG that is higher and wider than the IOM guideline.

There are some limitations to our study. First, this is a retrospective study. No reliable and objective method was used to measure the pre-pregnancy BMI and GWG of the patients because they were calculated using pre-pregnancy body weight reported by each individual. Second, there was lack of birth weight curve adjusted on Korean population. The study population showed high rate of SGA and low rate of LGA stemming from ethnic differences. Third, several outcomes such as GDM, Apgar score, and admission to NICU were excluded from the most meaningful perinatal outcomes. Although we did not found statistically significant correlations in this study, it remains unchanged that they are important factors associated with GWG. Finally, the high cesarean section rate, which was a characteristic problem of Korean population, could affect the results of the statistical analysis. Despite such limitations, our study has strength in that it provided a reference standard for clinical practice by presenting revised GWG guidelines suitable for Korean population. However, further prospective studies required to determine both adverse pregnancy outcome depending on high GWG and optimal GWG for Korea population. There is a need to conduct prospective studies based on the Korean birth weight standard in the future.

## Conclusions

There are many factors that affect perinatal morbidity and mortality, but a typical modifiable influencing factor is maternal weight gain during pregnancy [25, 26]. Pregnant women who gain weight according to the recommend guidelines have a good prognosis for birth weight, fetal growth, and postpartum weight retention [27]. Therefore, it is an important task of healthcare providers to improve maternal health by proposing and managing appropriate GWG. Furthermore, GWG guidelines adapted to the characteristics of the Asian population need to be considered, although the IOM guideline is the most used currently.

## Abbreviations

BMI: Body mass index
GWG: Gestational weight gain
IOM: Institute of Medicine
LGA: Large for gestational age
PTB: Spontaneous preterm births
SGA: Small for gestational age
WHO: World Health Organization
